# Life of D. Hays Agnew

**Published:** 1893-07

**Authors:** 


HISTORY OF THE LIFE OF D. HAYES AGNEW, M. D., LL. D. By J. Howe Adams, M. D. With fourteen full-page portraits and other illustrations. In one large Royal Octavo volume, 376 pages, Extra Cloth, beveled edges, $2.50: Half-Morocco, gilt top, $3.50 net. *Sold only by subscription.* Philadelphia. The F. A. Davis Co., Publishers, 1914 and 1916 Cherry Street.

In presenting the history of the life and life-work of Dr. Agnew, Dr. Adams, the author, states that Dr. Agnew lived his life without thought of a biographer, consequently but little data for this work could be found among his papers. Under these circumstances the author has had to depend on Mrs. Agnew and the friends of Dr. Agnew for the material necessary for such a work. This information was furnished at the expense of considerable toil, trouble and time, and it seemed to be a pleasant task on the part of all to do what they could for their beloved friend and colleague.

The author first gives the lineage of the Agnew family, beginning in the tenth or eleventh century. He then takes up the early life, the school life and early professional life of Dr. Agnew. He tells of how he began practice in a modest way in a small country village—of his abandoning the practice of medicine and engaging in a business venture—of his re-entering the practice and the many disadvantages under which he labored,—of his removal to Philadelphia, and of his connection with the Philadelphia School of Anatomy,—of his success in this field, and of his gradual rise from an obscure, unknown country doctor to the high and honorable position of Professor of Surgery in the University of Pennsylvania, a surgeon of the first rank, honored and respected by his colleagues, and beloved by all who knew him. Such is the life and history of one of the greatest surgeons that America has ever produced. This book will be read by thousands of physicians who love and respect the memory of Dr. Agnew, and Dr. Adams will receive the thanks of all of them for his earnest and painstaking efforts in producing such a history. H.

---

THE YEAR-BOOK OF TREATMENT FOR 1893. A Critical Review for Practitioners of Medicine and Surgery. A Series of Contributions by Twenty-two Writers. In one 12mo. volume of 500 pages. Cloth, $1.50. Philadelphia, Lea Brothers & Co., 1893.

It is almost unnecessary to point out the great usefulness of such a volume to every practitioner of any department of medicine. To keep apprised of all important advances is a binding obligation, but it cannot be fulfilled by individual reading. Even if it were still possible for one mind to scan the vast accessions to medical literature, it could not pass critical judgment upon the claimed advances in more than a few of the many special subjects essential to a thorough equipment. In *The Year-Book of Treatment* this double function is performed by a corps of acknowledged leaders, covering in their joint survey all branches of medicine, and qualified to select and present the real progress of the twelvemonth. The result of their labors is of a value so widely recognized that the demand for this work renders possible its presentation at a price within the reach of all. Its contents are carefully classified and indexed, and full references to original sources given for the benefit of those desiring to make further research. It would be difficult to imagine a book more closely suited to the needs of the medical practitioner or writer.

---

FERMENTATION, INFECTION AND IMMUNITY. By J. W. McLaughlin, M. D., Austin, Texas. Von Boeckmann, 1892. 240 pages, price, $2.50.

The aim and purpose of this essay is to show that the accepted principles of molecular physics, and those of chemistry and biology, if supplemented by legitimate deductions from them, are amply sufficient to account for all the known phenomena of these processes (fermentation, infection and immunity), and also to explain their relationship and intimate nature." Such in the author's own words, is the object of his book. This "physical theory," as, for want of a better name, he calls it, will, according to the writer, explain all that previous hypotheses have made clear, and in addition, much that they have left in obscurity concerning the phenomena of fermentation, etc.

He writes upon the subject evidently *con amore* and has given us a well digested monograph, in which he discusses most of the recent work of Metschnikoff and other investigators in bacteriology and kindred branches of science.

Whether the physical theory will oust or supplement prior hypotheses regarding immunity, time alone will tell. Be that as it may, Dr. McLaughlin's essay is well worth perusal.—[*From British Medical Journal*, Saturday, April 8, 1893, page 744.

